# Analysis of conserved miRNAs in cynomolgus macaque genome using small RNA sequencing and homology searching

**DOI:** 10.7717/peerj.9347

**Published:** 2020-07-10

**Authors:** Xia Huang, Shijia Li, Xiaoming Liu, Shuting Huang, Shuang Li, Min Zhuo

**Affiliations:** 1 School of Biology and Biological Engineering, South China University of Technology, Guangzhou, Guangdong, China; 2 Guangzhou Tulip Information Technologies Ltd., Guangzhou, Guangdong, China

**Keywords:** microRNA, RNA sequencing, Cynomolgus macaque

## Abstract

MicroRNAs (miRNAs) are important regulators that fine-tune diverse cellular activities. Cynomolgus macaques (*Macaca fascicularis*) are used extensively in biomedical and pharmaceutical research; however, substantially fewer miRNAs have been identified in this species than in humans. Consequently, we investigated conserved miRNA profiles in cynomolgus macaques by homology searching and small RNA sequencing. In total, 1,455 high-confidence miRNA gene loci were identified, 408 of which were also confirmed by RNA sequencing, including 73 new miRNA loci reported in cynomolgus macaques for the first time. Comparing miRNA expression with age, we found a positive correlation between sequence conservation and expression levels during miRNA evolution. Additionally, we found that the miRNA gene locations in cynomolgus macaque genome were very flexible. Most were embedded in intergenic spaces or introns and clustered together. Several miRNAs were found in certain gene locations, including 64 exon-resident miRNAs, six splice-site-overlapping miRNAs (SO-miRNAs), and two pairs of distinct mirror miRNAs. We also identified 78 miRNA clusters, 68 of which were conserved in the human genome, including 10 large miRNA clusters predicted to regulate diverse developmental and cellular processes in cynomolgus macaque. Thus, this study not only expands the number of identified miRNAs in cynomolgus macaques but also provides clues for future research on the differences in miRNA repertoire between macaques and humans.

## Introduction

MiRNAs are small (18–25 nucleotides long), non-coding RNAs that regulate the expression of target genes at the post-transcriptional level. Generally, miRNA genes are transcribed by RNA polymerase II and form primary miRNA hairpins (pri-miRNAs). A region of a pri-miRNA forming hairpin structure is recognized by a microprocessor complex consisting of Drosha and DGCR8 proteins, which produce a stem-loop precursor miRNA (pre-miRNA) (Lee et al., 2003). Pre-miRNAs are then exported to the cytoplasm and are further processed by the enzyme Dicer, which cleaves off the terminal loop, leaving a miRNA duplex about 22 nucleotides in length. Many hairpins produce functional mature miRNAs from both duplex arms (Kobayashi & Tomari, 2016; Rorbach, Unold & Konopka, 2018). Mature miRNAs assemble with Argonaute proteins into miRNA-induced silencing complexs (miRISCs) to mediate mRNA targeting, primarily through pairing between the miRNA seed region (nucleotides 2–8) and complementary site within the 3′  UTR of mRNA (Berezikov, 2011). In addition to the seed region, certain sites in the 3′  part of miRNAs can also contribute to target recognition (i.e., nucleotides 13–16 known as the ‘supplemental region’) (Gebert & MacRae, 2019). In animals, miRISCs induce gene silencing through translation repression and mRNA decay (Jonas & Izaurralde, 2015; Treiber, Treiber & Meister, 2019). A single miRNA usually regulates a large number of genes simultaneously, and one gene may be regulated by multiple miRNAs. In particular, transcription factors or RNA-binding proteins can be regulated by individual miRNAs and entire cellular pathways can be regulated by miRNA clusters. MiRNA binding of neighboring target sites on a target mRNA can result in cooperative repression (Gebert & MacRae, 2019; Uhlmann et al., 2012). It has been estimated that about 60% of mammalian protein-coding genes are conserved targets of miRNAs (Friedman et al., 2009). The comprehensive interactions between miRNAs and mRNAs often play significant roles in composing complex genetic networks and fine-tuning diverse biological functions, such as organism development, body patterning, tissue homeostasis, differentiation, and cell cycle regulation (Berezikov, 2011; Pinhal et al., 2018). Evolutionarily, many miRNAs are highly conserved due to their functional importance. However, excessive lineage-specific miRNAs have also emerged in various taxa (Heimberg et al., 2008; Bartel, 2018). It is worth noting that miRNA expansion appears to be associated with body-plan innovations and other phenotypic changes in bilaterians and vertebrates (Heimberg et al., 2008; Meunier et al., 2013; Christodoulou et al., 2010). Therefore, they contribute considerably to phenotypic evolution in animals.

Cynomolgus macaques (*Macaca fascicularis*), also known as crab-eating or long-tailed monkeys, are distributed widely in Southeast Asia, including Indonesia, Vietnam, Philippines, Cambodia, Myanmar, Malaysia, Brunei, and Thailand (Shiina et al., 2015). The species belongs to the Cercopithecinae subfamily of Old-world monkeys and has emerged as an alternative animal model after the banning of rhesus macaques (*Macaca mulatta*) from India. Cynomolgus macaques have been utilized extensively in biomedical and pharmaceutical research, including studies on aging, neurological diseases, diabetes, pregnancy, and drug evaluation (Theriault et al., 1999; Carter, 2011; Grigsby, 2016; Yang et al., 2014; Willard & Shively, 2012), thus prompting the sequencing of its genome (Ebeling et al., 2011; Yan et al., 2011). Based on comparative genomic analysis, cynomolgus macaques share 99.2% and 92.8% genomic identity with rhesus macaques and humans, respectively (Veeranagouda et al., 2015). Furthermore, recent animal study indicates a strong correlation between increasing metazoan morphological disparity and miRNA repertoires, although no correlation with protein domain diversity (Deline et al., 2018). Species-specific miRNAs are considered to be important contributors to cancers, autoimmune diseases, and dissimilar responses of species to toxicant exposure (Koufaris & Gooderham, 2013; Koufaris, 2016; Jimenez & Piera-Velazquez, 2013). Therefore, elucidating the differences in miRNA repertoires between monkeys and humans could have valuable implications for the use of nonhuman primate models in biomedical studies. So far, larger-scale annotations of miRNAs have only been conducted for a few species (Dannemann et al., 2012a; McCreight et al., 2017) and only 20 of the ∼300 known primate species have entries in miRBase (Kozomara, Birgaoanu & Griffiths-Jones, 2019; Perelman et al., 2011). Furthermore, the number of characterized human pre-miRNAs (*n* = 1, 917) is three times larger than that of rhesus macaques (*n* = 617). In addition, cynomolgus macaque miRNAs remain limited to several hundred (Yang et al., 2014; Veeranagouda et al., 2015; Yang et al., 2012). Thus, further studies are needed to extend the miRNA profile of this species. Recently, technological advances such as bioinformatics and high-throughput RNA sequencing allowed the identification of a great number of putative miRNAs in different organisms. The computational methods are better to discover conserved miRNAs comprehensively (Demirci, Baumbach & Allmer, 2017), while the RNA-seq method is more advantageous in identifying novel miRNAs with non-canonical structures. In this study, we used the two complementary approaches mentioned above to identify miRNAs in cynomolgus macaques. This study should not only expand the number of recognized miRNAs in cynomolgus macaques but also provide clues to elucidate the differences in miRNA repertoires between macaques and humans, and thus improve our understanding of the evolution and function of miRNAs.

## Material and Methods

### Sample collection

Peripheral whole blood samples were taken from four unrelated Vietnamese-origin cynomolgus macaques (2–4 years old, one male, three females). The monkeys were healthy and housed at Guangdong Landau Biotechnology Co., Ltd. (Guangzhou China). All experiments were reviewed and approved by the Institutional Animal Care and Use Committee (IACUC) of Guangdong Landau Biotechnology Co., Ltd. (project number: IACUC-003). Peripheral whole blood samples (2 ml) were used for small RNA extraction. Firstly, red blood cells were selectively lysed using an EZNA Blood RNA Kit (Omega, USA) and white blood cells were collected by centrifugation (450 g, 10 min, 4 °C). To avoid interference of 18S and 28S rRNA, small RNAs (18–200 nt) were isolated from white blood cells using RNAiso (TaKaRa, China). The samples were dissolved in 30 µl of diethylpyrocarbonate-treated water and stored at −80 °C prior to small RNA sequencing at the Beijing Genomics Institute (BGI, Shenzhen, China).

### Small RNA library construction and sequencing

Four small RNA libraries were prepared and sequenced using standard methods at BGI. Before library construction, the integrity and concentration of samples were checked using an Agilent 2100 Bioanalyzer (Agilent RNA 6000 Nano Kit, USA). The RNAs were size-fractionated on a 15% PAGE gel, and small fragments (range of 18–30 nt) were collected and then amplified by real-time polymerase chain reaction (RT-PCR) using adaptor primers for 17 cycles. The fragments (140–160 bp in length) were then fractionated with a 15% PAGE gel. The final library was quantified by determining average sequence length using the Agilent 2100 Bioanalyzer (Agilent DNA 1000 Reagents, USA) and quantifying the library using quantitative RT-PCR (StepOnePlus Real-Time PCR System, Applied Biosystems, Foster, USA). The fragment sizes of the four libraries were all between 140 bp and 160 bp and the concentration of each library was more than 8.5 nM, thus meeting the minimum requirements for the construction of a sequencing library. Finally, the libraries were used for sequencing analysis on an HiSeq 4000 System (Illumina, USA). The primary data were then subjected to low-quality filtering. Reads that satisfied any of the following parameters were removed: (1) reads aligned to adaptors or primers with less than three mismatches, (2) reads with >10% unknown bases (N bases), (3) reads with >50% low-quality bases in one read, and (4) reads <18 nt. The remaining clean reads were used in the following analyses. The clean reads of the four samples were submitted to the European Nucleotide Archive (ENA) under study accession number PRJEB38080.

### Analysis of sequencing data

The cynomolgus macaque reference genome (v. macFas5) was downloaded from the UCSC Genome Browser (http://genome.ucsc.edu). Clean reads were aligned to the reference genome using BGI software SOAP2 (Li et al., 2009b). Sequences perfectly mapped to the genome along their entire length were further annotated by alignment against macaque genome annotations and known annotation documents from the NCBI/UCSC/Ensembl databases (Benson et al., 2013; Kalvari et al., 2018). Sequences matching rRNA, tRNA, snRNA, snoRNA, and repeat associated sequences or overlapping with exons were discarded, and the remaining sRNA tags were aligned to a reference set of all animal miRNAs from miRBase (release 22) using BLAST by allowing no more than two mismatches outside the seed region. To distinguish authentic miRNAs from false annotations, candidates corresponding to known miRNAs from miRBase were considered conserved miRNA orthologs in the cynomolgus macaques when they were supported by at least 10 reads of mature sequences from the four samples and detected in at least three individuals according to criteria described previously (Veeranagouda et al., 2015; Kozomara & Griffiths-Jones, 2014). The pre-miRNA sequences were predicted based on the presence of hairpin structures analyzed by MIREAP under default settings (https://sourceforge.net/projects/mireap/) (Tan et al., 2013).

### Detection of miRNA genes by homology searching

BLAT and BLAST softwares were used for homology searching (Kent, 2002). All mammalian pre-miRNA sequences from miRBase (release 22) were aligned to the cynomolgus macaque genome. The candidates were assumed to be conserved pre-miRNA sequences in cynomolgus macaques only when they fulfilled three criteria: (1) similarity of full length pre-miRNA >93%; (2) gene location corresponding to only one miRNA (although can have multiple gene mapping information); and (3) in mature sequences, no more than two mismatches outside the seed region and no more than one mismatch in the seed region compared with existing miRNAs (Veeranagouda et al., 2015). Furthermore, 10 nt up and downstream of the conserved pre-miRNA sequences were analyzed by MIREAP (Tan et al., 2013) and RNAfold (Lorenz et al., 2011). Stem-loop hairpins were considered acceptable only when they fulfilled three criteria: (1) the mature miRNA present in one arm of the stem; (2) absent of large internal bulges (*n* < 6); and, (3) a minimal folding free energy (MFE) of predicted pre-miRNA secondary structures of no more than -15 kcal/mol. After mapping all pre-miRNA candidates with acceptable secondary structures to the genome, they were further annotated by alignment against known annotation documents from the NCBI/UCSC/Ensembl databases (Benson et al., 2013; Kalvari et al., 2018). Small RNAs were sorted by their chromosomal locations on the genome. Based on criteria described previously (Marco et al., 2013), if two neighboring miRNA loci were located within 10 kb and on the same strand, they were considered clustered miRNA genes. The miRNA orthologs were grouped into different miRNA families according to sequence similarity to known miRNA family members. ClustalW was used for the alignment of sequences. Data patch processing was implemented in python.

### Target prediction

The target genes of miRNAs were predicted by TargetScan 7.2 (http://www.targetscan.org), miRanda (http://www.microrna.org), and PITA (https://tools4mirs.org/). Gene ontology (GO) was applied for functional annotation analysis of the predicted targets. Enriched pathways involved with the miRNAs were determined according to the Kyoto Encyclopedia of Genes and Genomes (KEGG). Both GO and KEGG analyses were performed using the clusterProfilers package in R (Yu et al., 2012), and an adjusted *P*-value (Benjamini–Hochberg false discovery rate correction) of less than 0.05 was considered significant.

### Correlation between age and miRNA expression

Considering the variable RNA concentrations and biases in the RNA-seq procedures for each sample, the expression levels of mature miRNAs were normalized by transcripts per million, i.e., dividing the number of reads for each miRNA by the total number of clean reads for each sample and multiplying it by 10^6^ (Xu et al., 2013). The ages of the miRNAs were assessed based on the set of homologous sequences identified in other vertebrate species in miRBase (Meunier et al., 2013). If the miRNA homologous sequences were shared among vertebrates or mammals, these miRNAs were considered old and grouped into vertebrate or mammalian categories, respectively. If the miRNAs were specific to primates or Cercopithecidae, they were considered young and grouped into primate or Cercopithecidae categories, respectively. The average expression levels of miRNAs in different age groups of the four cynomolgus macaques were statistically analyzed by GraphPad Prism 8. Spearman correlation coefficients (R values) between the age of miRNAs and average expression levels of mature miRNAs were computed by matrix analysis in GraphPad and *P*-values were derived at a confidence interval of 95%.

## Results

### Identification of miRNA genes in cynomolgus macaque genome by homology searching

To identify previously unknown miRNA sequences in macaques (Yang et al., 2014; Veeranagouda et al., 2015), we used all mammalian pre-miRNA sequences deposited in miRBase as a reference for homology searching. According to the criteria described above, a total of 1,697 homologous sequences were perfectly mapped to the cynomolgus macaque genome with nonoverlapping gene locations and were then used to analyze their secondary structures. 1,526 of the 1,697 homologous sequences were predicted to form acceptable stem-loop hairpins and were considered conserved miRNA candidates. Additionally, we annotated the 1,526 candidates in the cynomolgus macaque genome using the NCBI/UCSC/Ensembl databases and miRBase for screening of homologies in other species. A candidate identified by homology searching was deemed to be of high confidence if it met any of the following conditions: (1) annotated as miRNA in other species or homolog has experimental support; and, (2) reported as a *Macaca fascicularis* miRNA previously. In total, 1,455 of the 1,526 pre-miRNA candidates showed high confidence. The remaining 71 pre-miRNA candidates lacked support in the other species and were excluded from the following analysis. 672 of the 1,455 candidates were reported previously and the remaining 783 gene loci were firstly annotated in cynomolgus macaque genome (Yang et al., 2014; Veeranagouda et al., 2015). Compared the 1,455 pre-miRNAs with their homologs in other mammals, the stem-loop sequence similarities of 1,186 candidates (81.5% of 1,455) exceeded 95%. Furthermore, 1,074 candidates (73.8% of 1,455) contained 0 mismatches in the -5 p or -3 p mature sequences, with the remaining 299 and 82 having one and more than one mismatch, respectively. In comparison to human miRNAs, 1,083 of the 1,917 human pre-miRNAs deposited in miRBase had orthologs in the cynomolgus macaque genome. Thus, most pre-miRNA and mature miRNA sequences were highly conserved. Most of the 1,455 miRNA sequences in the cynomolgus macaque were single copies, although others had paralogs at several gene locations. For instance, mir-548, a poorly conserved primate-specific miRNA gene family, was represented by 53 paralogs distributed across 18 chromosomes, similar to that observed in humans (Liang, Guo & Liu, 2012). The names, stem-loop sequences, gene locations, homology similarities, mismatches in mature sequences, and MFE values of the predicted hairpin structures for the 1,455 pre-miRNA candidates are listed in Table S1.

### Identification of miRNAs in cynomolgus macaque using small RNA sequencing

A major obstacle in research on primate miRNAs is the lack of experimentally verified miRNAs in nonhuman primates. To better characterize the identified miRNAs in cynomolgus macaques, small RNA-seq was performed using peripheral blood leucocytes from the four animals. On average, 14 million clean reads were obtained and about 9 million small RNAs (∼5,000 unique reads) were mapped to miRBase for each sample (Table 1). As described in the methods, sequences that matched against orthologs in miRBase were considered as known miRNAs, otherwise, they were considered novel. A total of 546 known mature miRNA sequences and 136 potentially novel mature miRNAs (Table S2) were identified. As the 136 potentially novel miRNAs need further confirmation, only the 546 known miRNAs were used in subsequent analyses. To understand whether these miRNAs resulted from one or both of arms of a given precursor, we assigned the 546 mature miRNAs based on pre-miRNA information. As shown in Table S3, 337 mature miRNAs were expressed from both the -5 p and -3 p arms of 170 pre-miRNAs, and the remaining 209 mature miRNAs were expressed from either the -5 p or -3 p arms of 213 pre-miRNAs. As seven mature miRNAs were derived from more than one pre-miRNA, the 546 mature miRNAs were processed from 383 stem-loop miRNA sequences. We identified 379 of the 383 pre-miRNAs by homology searching (as above), including 357 pre-miRNAs with mapping information for single gene and 22 with mapping information for 2–4 genes. We re-screened the remaining four pre-miRNAs, mir-383, mir-378c, mir-4536, and mir-5698, which were deposited in miRBase against the cynomolgus macaque genome. All showed sequence similarities below 60%, indicating that these four pre-miRNA sequences were distinct to cynomolgus macaques. Analysis of sequencing data indicated that 408 of the 1,455 miRNA loci were expressed in peripheral blood leucocytes. These 408 miRNA loci demonstrated high confidence as they were identified by both RNA sequencing and homology searching and showed homolog support in other species. In total, 335 have been reported in cynomolgus macaques previously (Yang et al., 2014; Veeranagouda et al., 2015), with the remaining 73 considered to be new pre-miRNA loci in cynomolgus macaques (Table 2).

**Table 1:** Clean reads of RNA sequences in cynomolgus macaques mapped to mature miRNAs in miRBase.

	CE01	CE02	CE03	CE04	Average
Clean reads	11640333	10922961	11573957	20255682	13598233
Unique sRNAs	365682	469182	426731	219056	370163
Clean reads for miRNAs	7686565	6746516	6772031	15351472	9139146
Unique sRNAs for miRNAs	5845	4705	5319	4116	4996

**Notes.**

CE01, CE02, CE03, and CE04 represent four unrelated cynomolgus macaques. Clean reads are total sRNA reads after filtering noise. Unique sRNAs represent numbers of sRNA types. Clean reads for miRNAs mean clean reads mapped to known miRNAs in miRBase.

**Table 2:** Seventy three newly identified pre-miRNA genes loci in macaque genome.

pre-miRNAs	Accession numbers	Sequences	Average expression level	
			-5p	-3p
mir-10399	LR745789	AATTACAGATTGTCTCAGAGAAAACAGATGAGTTACTCTCTCAGACAAGCTGTAGGTC	14.04	
mir-1246	LR594712	TGCATCCTTG AATGGATTTTTGGAGCAGGAGTGGACACCTGACCCAAAGGAAATCAATCCATAGGCTAGCAAT	0.96	
mir-1248	LR594713	TTT ACCTTCTTGTATAAGCACTGTGCTAAAATTGCAGACACTAGGACTATGTCTTGGTTTTTGCAATAAT GCTAGCAGAGTACACACAAGAAGAAAAGTAACAGCA	2.55	
mir-124-3	LR745790	TGAGGGCCCCTCTGCGTGTTCACAGCGGACCTTGATTTAATGTCTATACAA***TTAAGGCACGCGGTGAATGCCA*** AGAGAGGCGCCTCC		0.22
mir-1294	LR594714	CACTTAATATGTGCCAAGATCTGTTCATTTATGATCTCACTGAGTCC TGTGAGGTTGGCATTGTTGTCT GGCGTTGTCTGATATACAACAGTGCCAACTTCACAGGACTCAGTGAAGTGAAGCTGAGGATTAGGAAGGTGTG	1.04	
mir-1301	LR594715	CTGCCAAGCGACCCCTAGAATGGGGATTGTGGGGGGTCGCTCTAGGCACCGCAGCACTGTGCTGGGGATG *** TTGCAGCTGCCTGGGAGTGACTTC*** ACACAGTCCTCTCTGCCT		30.95
mir-1307	LR594716	CATCAAGACCCAGCTGAGTCACTGTCACTGCCTACCAATC TCGACCGGACCTCGACCGGCT CGTCTGTGTTGCCAATCG ***A*CTCGGCGTGGCGTCGGTCGTG**GTAGATAGGCGGTCATGCATACGAATTTTCAGCTCTTGTTCTGGTGAC	1.02	543.87
mir-1327	LR745791	GGGGACTGCTCTT TAAAAGGCTGTTAAATGGTGAT ACTATATTTTTTAACATCAGCATCATTTAGAAACCTTTTAGAGAACAGTTCTC	1.08	
mir-138-1	LR745792	AGCTGGTGTTGTGAATCAGGCCG TTGCCAATCAGAGAACGGCTACTTCACAACACCAGGGCC	2.20	
mir-1468	LR745793	GGCGGGCGGTTT CTCCGTTTGCCTGTTTTGCTGA TGTACATTCAACTCATTCTCAGCAAAATAAGCAAATGGAAAATTTGTCCATC	3.87	
mir-151b	LR594718	ACCTCTGATGTATCAATCTCTCTTCGGGGCTCCCGAGACACAGAAACAGACACCTGCCC *** TCGAGGAGCTCACAGTCT*** AGACAAACAAACCCAGGGT		3.50
mir-1839	LR745794	GAAAAGGTAGATAGAACAGGTCTTGTTTGCAAAATAAACTC***AAGGCCTACTTATCTACCAAC***AG	249.11	1.85
mir-1843	LR745796	TATGGAGGTCTCTGTCTGGCTTAGGACAGCTGGCTAAG ***TCTGATCGTTCCCCTCCATAC***A	33.89	51.60
mir-1976	LR745798	TGGCCTCTGGGCACGGGGGTTGGGTGTGCAAAGGGTGGCAGCAAGGAAGGCAGGGTTCCTGAGGTGTGTC CTCCTGCCCTCCTTGCTGTAGACTTTGGCCTGAGCAAGGAG	1.60	
mir-199b	LR594719	CCAGAGGACACCTCCACTCCGTCTA CCCAGTGTTTAGACTATCTGTTC AGGACTCCCAAATTGTACAGTAGTCTGCACATTGGTTAGGCTGGGCTGGGTTAGACCCTCGG	230.72	
mir-200b	LR745799	CCAGCTCGGGCAGCCGTGGC CATCTTACTGGGCAGCATTGGA TGGAGTCAGGTCTC *** TAATACTGCCTGGTAATGATGA*** CGGCGGGGCCCTGCACG	0.79	13.71
mir-219b	LR594722	GGAGCTCAGCCAC AGATGTCCAGCCACAATTCTCG GTTGGCCGCAGACTCGTACAAGAATTGCGTTTGGACAATCAGTGGCGAAGCCC	1.33	
mir-2355	LR594723	ATCCCCAGATACAGTGGACAATATGCTATTATAATTGTATGGC *** ATTGTCCTTGCTGTTTGGAGAT*** AA		10.11
mir-3074	LR594725	GGGCTCGACTCCT GTTCCTGCTGAACTGAGCCAG TGTGTAAAATGAGAACTGATATCAGCTCAGTAGGCACCGGAGGGCGGGTCC	11.20	
mir-3074-2	LR745800	CAGGCTCCAAGGGGGCTTGACTCCT GTTCCTGCTGAACTGAGCCAG TGTGCACAAACCAACTGTGTTTCAGCTCAGTAGGCACGGGAGGCAGAGCCCAGGGAGGCCA	11.20	
mir-30e	LR745801	TTCTGGGCAGTCTTTGCTAC TGTAAACATCCTTGACTGGAAG CTGTAAGGTGTTCAGAGGAGC ***T*TTCAGTCGGATGTTTACAGC** GGCAGGCTGCCACGGTCGTCCCCAGCTAC	3875.85	1023.80
mir-3120	LR594726	GGCTGGGTTGTCATGTGACTG CCTGTCTGTGCCTGCTGTACA GGTGAGCGGATGTTCTG *** CACAGCAAGTGTAGACAGGCA*** GACACATGACAACTCTGTCCAGCC	4.30	9.52
mir-3155a	LR745803	CACTTTTGAGACGCCTGTTCCGGGCATCA GCTCCCACTGCAGAGGCTGG GGAGCCGGACAGCTCCCTTCC *** CAAGCTCTGCAGTGGGAACTGA*** TGCCTGGAACAGTTCCTGCA	0.44	7.83
mir-3158	LR594729	ATTCAGGCTGGTCCTGCAGAGAGGAAGCCCTTCTGCTTCCAGGTATTGG*** AAGGGCTTCCTCTCTGCAGGAC*** CAGCCTGAAT		14.74
mir-3158-2	LR745804	ATTCAGGCTGGTCCTGCAGAGAGGAAGCCCTTCCAATACCTGGAAGCAG *** AAGGGCTTCCTCTCTGCAGGAC*** CAGCCTGAAT		14.74
mir-3160-1	LR594730	GGACCTACCCTGGGCTTTCTAGTCTCAGCTCTCCTCCAGCTCAACTGGTCAGG *** AGAGCTGAGACTAGAAAGCCCA*** GGGCAGGTTC		0.72
mir-3160-2	LR745805	ACCTGCCCTGGGCTTTCTAGTCTCAGCTCTCCTGACCAGTTGAGCTGGAGG *** AGAGCTGAGACTAGAAAGCCCA*** GGGTAGGT		0.72
mir-3164	LR745806	CTTGGAAAC TGTGACTTTAAGGGAAATGACG CACAGCAGGCCCTGGAATCACG *** CCGTTTTGCTTGAAGTTGCAGT*** TTCCCAGG	0.97	0.56
mir-3174	LR745808	CCAGCATCAGCATTACCTGG TAGTGAGTTAGAAATGCAGAGC CCCGGGCTTCTCAGCAAACCTACTGGATCTG CATTTTAATTCACATGCATGGTAATGTCTGTAAAGCACT	3.28	
mir-3191	LR745809	GGGGTCACCTGTCTGGCCGTCTACCTTCCACACTGACAAGGGCCG *** TGGGGACGTAGCTGGCCAGACAG*** GTGACCCC		1.24
mir-320b-1	LR745810	AATTAATCCCTCTCTTTCTAGTTCTTCCTAGAGTGAGG *** AAAAGCTGGGTTGAGAGGGCAA*** ACAAATTAACTAATTAATT		119.04
mir-320c-1	LR745811	AAAAATGAGGCCTTATCTTCCCAGTTCTTCCCAGAGTCAGG *** AAAAGCTGGGTTGAGAGGGT*** AGAAAAAAAAT		41.88
mir-320d-1	LR745812	TTCTCTTCCCAGTTCTTCCCAAAGTTGAG *** AAAAGCTGGGTTGAGAGGA***		25.86
mir-320d-3	LR745813	TCTCTTCCTGGTTCTTCCCGAAGTCAGG *** AAAAGCTGGGTTGAGAGGA***		25.86
mir-320e	LR745814	CTCCATGGGGCTTTCTCTTCCCAGTTCTTCCTGGAGTCGGGG *** AAAAGCTGGGTTGAGAGGGTGA*** ACAGAAAAA		2.08
mir-326	LR594733	CTCATCTGTCTGTTGGGCTGGAGGCAGGGCCTTTGTGAAGGCG GGTGGTGCTCAGATCG *** CCTCTGGGCCCTTCCTCCAG*** CCCCGAGGCGGATTCA		7.38
mir-3609	LR594734	GTAACATTAACTTTTATTCTCGTTTTCCTTTTCTCTACCTTGTAGAGAAG *** CAAAGTGATGAGTAATACTGGCTG*** GAGCCC		1.38
mir-3613	LR745815	TGGTTGGGTTTGGAT TGTTGTACTTTTTTTTTTGTTC GTTGCATTTTTAGGAACAAAAAAAAAAGCCCAACCCTTCA CACCACTTCA	115.70	
mir-3620	LR745816	GTGAGGTGGGGGCCAGCAGGGA GTGGGCTGGGCTGGGCTGGGCC AAGGTACAAGGCCTCACCCTGCATCCCGCACCCAG	0.81	
mir-3691	LR594736	TTGAGGCACTGGGT AGTGGATGATGGAGACTCGGTAC CCACTGCAGAGGGTGGGGACCAAGTCTGCATCAT CCACTCCTCAGTGCCTCAG	0.89	
mir-371b	LR745817	GGTAAC ACTCAAAACATGGCGGCACTT TTTTCACCAGAGAGCAGAAAGTGCCCCCACAGTTTGAGTGCC	1.38	
mir-378g	LR745818	C ACTGGGCTTGGAGTCAGAAG ACCTGACTCCAGCCCAGGTC	0.40	
mir-3913-1	LR745819	TTGTTTATAATAAACTGAAATA TTTGGGACTGATCTTGACACTCT TACATAAAATGTTTTGGCAGACATCAA GATCAGTCCCAAATATTTCAGTTTATTATAGACAG	8.91	
mir-4485	LR594738	AGAGGCACCGCCTGCCCAGTGACACATGTT *** TAACGGCCGCGGTACCCTGA*** CTGTGCA		0.38
mir-4659a	LR745820	GAAACTGATGAAGCTGCCATGTCTAAGAAGAAAACTTTGGAGAAAAAT *** TTTCTTCTTAGACATGGCAGCG*** TCAACAGTTTC		0.93
mir-4661	LR745821	TTTACTCTG *** AACTAGCTCTGCGGATCCTGAT*** AGACAGCCTGATAGACAGTATCCACAGAGCTAGTCCAGAGTAAA	0.75	
mir-4676	LR594739	TGAACAAAA GAGCCAGTGGTGAGACAGTGA GTTGATTACTTCTCACTGTTTCACCACTGGCTCTTTGGTTCA	16.80	
mir-4684	LR594740	GCACCAGGGGTAC CTCTCTACTGACTTGCAACATA CATTTGTATTGGTG *** TGTTGCAAGTCAGTGGAGAGGT*** ACCCTTGGTGT	0.71	0.56
mir-4791	LR594741	TAAGAAC TGGATATGAAGACTGAAA TAAGCTCCATATCAATGAGAATTTCAATGGGATTATGTATAGTCAAT GTCCAGTAATTA	0.63	
mir-486-1	LR745823	TCTCCATCCTCCCTGGGGCA TCCTGTACTGAGCTGCCCCGAG GCCCTTCATGCTGCCCAGCT *** CGGGGCAGCTCAGTACAGGAT*** ACCTCGGGGTGGGAGTCAGCAGGAGGTGA	7013.65	332.08
mir-4999	LR745825	ATAGAAAATAAAACACATACT GCCGTATTGTCAGGTAGTGATA GGATTTA *** TCACTACCTGACAATACAATAT*** GTGTTTGTTTTATTTTATGT	1.41	0.26
mir-548ad	LR594743	CTATTAGGTTGGTGC AAAAGTAATTGTGGTTTTTG AAAGTAACTTGGCGAAAACCACAATGACTTT GCACCAACCTAATAC	2.20	
mir-548at	LR745827	TAGGTTGGTG CAAAAGTTGTTGCGGTTTTGGC CGCCAAAAGAAATGGCCAAAACCGCAATAACTTTT GTACCAACCTAA	1.34	
mir-548av	LR594747	AAAAGTACTTGTGGATTTGCCATTACCTTTACCTTTAATGGC *** AAAACTGCAGTTACTTTTGC***		0.36
mir-548ay	LR594748	AGAAGATGCTTACTACTAGGTTGGTGC AAAAGTAATTGTGGTTTTTGC ATTTAAAGTAATGGCCAAAACCGCG ATTACTCTTGCACGAACCTAACGGTAACACTTCT	3.98	
mir-548l	LR745828	TATTAGGTTGGTGCAAAAGTATTTGCGGGTTTTGTCATTGAAAGTAATGG *** CAAAAACTGCAATTACTTCTGC*** ACCAACCTAATGC		1.08
mir-5690	LR594755	CTTTTAATT TCAGCTACTACCTCTATTAGG ATTTGGGAATTATACTAATAGAGGTAGTAGTTGAAATTAAGAG	4.21	
mir-610	LR594756	TCTATTTGTCTTAGG TGAGCTAAATGTGTGCTAGGA CACATTTGAGCCAAATGTCCCAGCACACATTTAGC TCACATAAGAAAAATGAACTCTAGT	8.26	
mir-641	LR594757	CAGGCTGGGTGAAAGGAAGG AAAGACATAGGATAGAGTCACCTC TGTCCTCTGTCCTCCACCTATAGAGGTGA CTATCCTATGTCTTTCCTTCCTCTCACCCCTGAGTCTCA	1.06	
mir-6501	LR594758	GG AGTTGCCAGGGCTGCCTTTGGT GACAGCAGCAGTAGAGTTGCCAGAGCAGCCTGCGGTAACAGTA	1.74	
mir-6503	LR745829	AATGGTCCCCCAGGG AGGTCTGCGTTCTAATCCCCA GAAGCTAAGGATTAGG *** GGGACAAGGATGCAGACCTCC*** CTGGGGGACCGTT	7.58	18.52
mir-6503-2	LR745831	AATGGTCCCCGAG GGAGGTCTGCATTCTAATC CCCAGAAGCGAAGGATTAGG *** GGGACAAGGATGCAGACCTCC*** CTGGGGGACCATT	0.17	12.61
mir-6516	LR745833	TGGGTTTTGAA TTTGCAGTAACAGGTGTGAGCA TTCTAGCAGCAGTTTGGTGATCATGTATGATACTGCAAACAGGACCTA	0.32	
mir-655	LR594759	TTCGTTTCAGAACTATTCAAGGATATTTGAGGAGAGGTTATCCGT GTTATGTTCGCTTCATTCATCATGA *** ATAATACATGGTTAACCTCTTT*** TTGAATATCAGACTCT		2.35
mir-6731	LR594760	ACAGG TGGGAGAGCAGGGTATTGTGGA AGCTCCAGGTGCCAACTGCCTGCCTCTATCCCCCACTCTCCCCAG	1.20	
mir-6735	LR745834	GCAGCC AGGGCAGAGAGCACAGGAATCTGA GGTGACTGGCACAGAAGACTC *** AGGCCTGTGGCTCCTCCCCCAG***	1.14	0.09
mir-6813	LR594761	ACAGG CAGGGGCTGGGGTTTCAGGTTCT CAGTCAGAACCTTGGCCCCTCTCCCCAG	0.69	
mir-6816	LR745836	CCGAGTGGGGCGGGGTGGGTCCCTGCAGGGACTGTGACACT *** GAAGGACCTGCACCTCCGCCCACA*** G		0.82
mir-6866	LR745837	CCATT TTAGAGGCTGGAATAGGGATT ATTGAGTCTGGAAGAGTAAGGATCCCTTTATCTGTCCTCTAG	0.69	
mir-7155	LR594762	TCTGGGGTCTTGGGCCATCTGGTTGTGACAGCCCCGA *** TGGCCCAAGACCTCAGACC***		0.68
mir-744	LR594763	TTGGGCAAGG TGCGGGGCTAGGGCTAACAGCA GTCTTACTGAAGGTTTCCTGGAAACCACGCACATG *** CTGTTGCCACTAACCTCAACCT*** TACTCGGTC	1140.67	1.71
mir-7848	LR594765	GCTGGAGCTGGGTGGGTGTGGCAGGCCCACCGTGGGTATGC AAAGCTCTGACAATGTTTTACTTG *** CTACCCTCGGTCTGCTTACCACA*** CTCCCAGTTCCAC		18.23
mir-935	LR745838	GGCGGGGGCGCGGGCGGCAGTGGCGGGAGCGGCCCCTC GGCCATCCTCCGTCTGC *** CCAGTTACCGCTTCCGCTACCGC*** CGCCGCTCCCGCT		1.44

**Notes.**

Average expression levels for each mature miRNA were normalized by transcripts per million. Single or double underlines indicate mature miRNAs expressed from -5 p or -3 p arms of pre-miRNAs, respectively.

**Figure 1:**
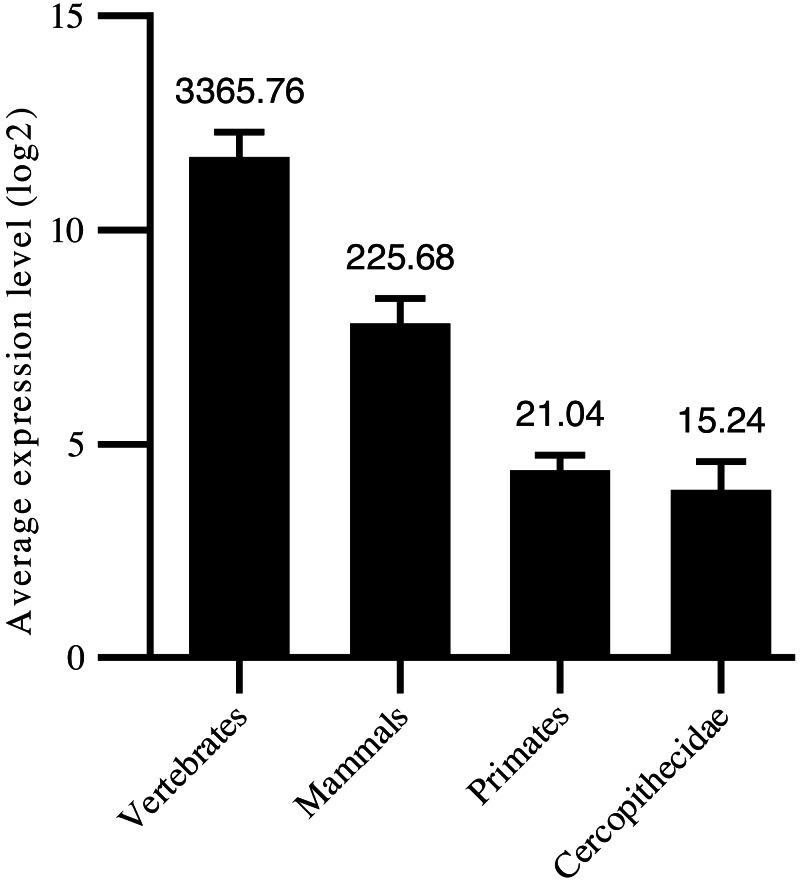
Age of mature miRNAs relative to expression levels. Primate-specific miRNAs showed approximately 10-fold and 160-fold lower average expression levels than ancient miRNAs from mammals and vertebrates, respectively.

Thus, in the current study, we confirmed 1,455 high-confidence pre-miRNA sequences by homology screening and RNA sequencing. According to their sequence similarities (Dostie et al., 2003; Yue, Sheng & Orwig, 2008; Bartel, 2009), 915 were grouped into 491 families (Table S1). Another 540 were not clustered into currently known families, suggesting new targeting properties. To assess the age of the miRNAs, their precursor sequences were searched in miRBase. The age of miRNAs was based on the group of species in which miRNA homologous sequences were identified. We classified the 1,455 miRNAs into four groups: i.e., 267 conserved in vertebrates, 332 conserved in mammals, 806 primate-specific, and 50 Cercopithecidae-specific. Among the 408 high-confidence pre-miRNA sequences, 179 were conserved in vertebrates, 140 were conserved in mammals, 77 were conserved in primates, and 12 were conserved in Cercopithecidae. We then examined the relationship between average expression level of miRNAs and their age and found a positive correlation (Spearman R: 0.27 to 0.42, *P* < 10^−4^; Fig. 1). Primate-specific miRNAs demonstrated lower average expression levels (10-fold and 160-fold, respectively) than ancient miRNAs in mammals and vertebrates. In addition, Cercopithecidae-specific miRNAs showed the lowest expression level among the four groups.

### Characteristics of miRNA gene locations in cynomolgus macaques

According to the annotations of the 1,455 high-confidence pre-miRNA sequences (Table S1), the miRNA encoding genes in the cynomolgus macaque genome were also distributed in diverse locations, similar to that found in other mammals (Kim & Kim, 2007; Morin et al., 2008). Of the 1,455 pre-miRNAs, approximately 46% were located in intergenic regions, 49% were located in introns and 5% were located in RNA coding regions. It is worth noting that 64 pre-miRNA sequences were processed from exons of protein-coding transcripts or non-coding transcripts, including 46 derived from untranslated regions. Six pre-miRNA sequences were found overlapping intron-exon junctions, i.e., SO-miRNAs. Most miRNA gene is encoded by one strand of one locus as above, but in rare cases, two distinct pre-miRNAs are found transcribed and processed on both strands of one locus (Tyler et al., 2008; Picao-Osorio et al., 2015; Mohammed et al., 2018). Two pairs of these so-called antisense or mirror miRNAs were identified in our study, i.e., miR-3120/miR-214 and miR-24-1/miR-3074 (Table S1). Here, miR-3120 was located within an intron of the dynamin-3 gene and miR-214 was embedded on the antisense strand of the same locus. For the miR-24-1/miR-3074 pair, we found that miR-24-1 was embedded within an intron of the *AOPEP* gene and miR-3074 was processed from the antisense strand. The miR-24-1/miR-3074 mirror miRNAs were first reported in this study in cynomolgus macaques (Cordero et al., 2015).

### MiRNA gene clusters in cynomolgus macaques

Previous studies have revealed that miRNAs are significantly enriched in clusters in introns or intergenic regions (Marco et al., 2013; Wang et al., 2016; Altuvia et al., 2005). To profile the clustering patterns of miRNAs in cynomolgus macaques, we grouped pre-miRNAs into distinct clusters following previous study (Marco et al., 2013). If two neighboring miRNA loci were located within 10 kb and on the same strand, they were considered to be clustered miRNA genes. Among the 1,455 pre-miRNAs, 285 miRNA genes were grouped into 78 distinct clusters. When comparing the 78 miRNA clusters to their human counterparts, if a human miRNA was not represented in the macaque clusters, it was screened cross the macaque genome in order to validate the existence of an ortholog in macaques (Wang et al., 2016). As such, 62 miRNA clusters were found to be similar to that in the human genome and six miRNA clusters showed minor changes (Table 3), indicating that most of the clustered miRNAs were evolutionarily conserved. Ten of the 78 clusters were newly emerged in the cynomolgus macaques. According to the similarity of seed regions of the miRNA sequences in each cluster, we grouped the 78 clusters into two sub-classes: 21 homo-seed clusters (miRNAs having identical sequences in the seed region) and 57 hetero-seed clusters (miRNAs having distinct sequences in the seed region). These results are consistent with previous studies suggesting that the composition of miRNA clusters is heterogeneous with a diversiform evolutionary mechanism (Wang et al., 2016). Homo-seed clusters are usually products of local duplications, and hetero-seed clusters are mainly shaped by a combination of *de novo* formations and local duplications (Wang et al., 2016).

**Table 3:** MiRNA gene clusters in cynomolgus macaques (*Mafa*).

Cluster in *Mafa*	miRNA number in *Mafa*	miRNAs in *Mafa*	Type	Detected by RNA-seq	Cluster in humans	miRNA number in humans	miRNAs in humans
chr1:128814989-128815918+	2	137/2682	hetero	no	chr1:98045270-98046113-	2	2682/137
chr1:186962061-186965108-	2	30c-1/30e	homo	yes	chr1:40754370-40757360+	2	30e/30c-1
chr1:227255265-227256419-	2	429/200a^a^	homo	yes	chr1:1167123-1169076+	3	200b/200a/429
chr1:30111736-30117576-	2	214/199a-2	hetero	yes	chr1:172138815-172144614-	2	214/199a
chr1:57185008-57185289-	2	181b-1/181a-1	homo	yes	chr1:198858886-198859130-	2	181b/181a
chr1:66367833-66368516-	2	29c/29b-1	homo	yes	chr1:207801864-207802513-	2	29c/29b-2
chr1:78854733-78855125-	2	215/194-1	hetero	yes	chr1:220117876-220118227-	2	215/194
chr10:30075091-30075493-	2	130b/301b	homo	yes	chr22:21652989-21653375+	2	301b/130b
chr10:31357983-31358388-	2	1306/3618	hetero	yes	chr22:20085796-20086129+	2	3618/1306
chr10:90709107-90709791-	2	296/298	hetero	yes	chr20:58817625-58818303-	2	296/298
chr11:112931512-112932150-	2	7705/10527^b^	hetero	no	not detected		
chr11:56821914-56822517-	2	26a-2/9947^b^	hetero	yes	not detected		
chr11:7274754-7275246+	2	200c/141	homo	yes	chr12:6963702-6964176+	2	200c/141
chr12:65115697-65116202+	2	7164/10b^b^	hetero	yes	not detected		
chr13:54048315-54054337+	2	216a/217	hetero	no	chr2:55983019-55989041-	2	217/216a
chr14:107474425-107475005+	2	34b/34c	homo	no	chr11:111512949-111513505+	2	34b/34c
chr14:6660857-6661975-	2	4691/7113	hetero	no	chr11:68032864-68033971+	2	7113/4691
chr15:108224172-108226742-	2	3596d/3596b^b^	homo	no	not detected		
chr15:10960721-10961048+	2	3154/199b	hetero	yes	chr9:128244744-128244977-	2	199b/3154
chr15:11398154-11399489-	2	181b-2/181a-2	homo	yes	chr1:198858886-198859130-	2	181b/181a
chr16:18135796-18136249-	2	6777/33b	hetero	no	chr17:17813479-17813916-	2	6777/33b
chr16:1954993-1955463-	2	132/212	homo	yes	chr17:2049928-2050350-	2	132/212
chr16:33365362-33370038+	2	10226/148c^b^	hetero	no	not detected		
chr16:43850674-43855488-	2	142/4736	hetero	yes	chr17:58331244-58336022-	2	142/4736
chr16:61839928-61840468-	2	3064/5047	hetero	yes	chr17:64500774-64501250-	2	3064/5047
chr16:7046633-7047042-	2	195/497	hetero	yes	chr17:7017627-7017999-	2	195/497
chr17:29263944-29264174-	2	16-1/15a	homo	yes	chr3:160404606-160404818+	2	15b/16-2
chr18:60473826-60477188+	2	1-2/133a-1	hetero	no	chr18:21825711-21829036-	2	133a/1
chr19:14206540-14206853+	2	181c/181d	homo	yes	chr1:198858886-198859130-	2	181b/181a
chr19:14522034-14522404+	2	8994/9307^b^	hetero	no	not detected		
chr19:2102695-2104529-	2	1227/6789	hetero	no	chr19:2234061-2235922-	2	1227/6789
chr2:104189414-104189980-	2	425/191	hetero	yes	chr3:49020158-49020694-	2	425/191
chr2:62802629-62802867+	2	15b/16-2	homo	yes	chr3:160404606-160404818+	2	15b/16-2
chr20:14704691-14710146+	2	193b/365a	hetero	yes	chr16:14303979-14309361+	2	193b/365a
chr20:44876971-44882123+	2	138/7181^b^	hetero	yes	not detected		
chr3:118484198-118485386+	2	489/653	hetero	no	chr7:93482783-93484006-	2	653/489
chr3:13210687-13210931-	2	6501/9922^b^	hetero	yes	not detected		
chr3:163632511-163633319-	2	29a/29b-2	homo	yes	chr1:207801864-207802513-	2	29c/29b-2
chr3:30725019-30725854-	2	let-7c/99	hetero	yes	chr21:16539100-16539904+	2	99a/let-7c
chr4:118507476-118512141-	2	133b/206	hetero	yes	chr6:52144400-52149009+	2	206/133b
chr6:148755825-148757619+	2	143/145	hetero	yes	chr5:149428943-149430720+	2	143/145
chr6:54606090-54606301-	2	449a/449b^a^	homo	yes	chr5:55170585-55172337-	3	449a/449b/ 449c
chr7:68810880-68814721+	2	1179∕7 − 2	hetero	no	chr15:88608120-88611917+	2	1179∕7 − 2
chr8:100682570-100682796-	2	599/875	hetero	yes	chr8:99536650-99536851-	2	599/875
chr8:136192174-136197663-	2	30b/30d	homo	yes	chr8:134800531-134804940-	2	30b/30d
chrun_ke145894:2386-3511-	2	10396a/663-10^b^	hetero	no	not detected		
chrx:111594304-111595504+	2	1912/1264	hetero	no	chrx:114651588-114652718+	2	1912/1264
chrx:144101311-144108532-	2	513b-1/513b-2	homo	no	chrx:147189716-147199114-	2	513c/513b
chrx:148922686-148923813-	2	224/452	hetero	no	chrx:151958583-151959699-	2	224/452
chrx:44130132-44131103-	2	221/222	homo	yes	chrx:45746179-45747094-	2	221/222
chrx:52628444-52629475-	2	98/let-7f-2	homo	yes	chrx:53556240-53557267-	2	98/let-7f-2
chrx:71471342-71471593-	2	421/374b	hetero	yes	chrx:74218391-74218608-	2	421/374b
chrx:71540122-71540378-	2	545/374a	hetero	yes	chrx:74287125-74287346-	2	545/374a
chrx:71841808-71842408-	2	7705/10527^b^	hetero	no	not detected		
chr10:4843233-4844250-	3	let-7b/**4763**/let-7a	homo	yes	chr22:46112751-46113766+	3	let-7a/4763/let-7b
chr14:118172242-118178007-	3	let-7a-2/ 10526 /100^a^	hetero	yes	chr11:122146522-122152296-	2	let-7a-2/100
chr15:108223782-108226749+	3	let-7a-2/let-7f/let-7d	homo	yes	chr9:94175961-94178916+	3	let-7a/let-7f-1/let-7d
chr15:109228189-109229083+	3	23b/27b/24-2	hetero	yes	chr9:95085226-95086085+	3	23b/27b/24-1
chr16:25123023-25123392-	3	451/144/4732	hetero	yes	chr17:28861376-28861722-	3	451a/144/4732
chr16:78700471-78708445-	3	657/338/1250	hetero	yes	chr17:81125290-81133285-	3	657/338/1250
chr19:14166279-14166650-	3	24-2/27a/23a	hetero	yes	chr9:95085226-95086085+	3	23b/27b/24-1
chr19:52420893-52421627+	3	99b/let-7e/125a	hetero	yes	chr19:51692617-51693327+	3	99b/let-7e/125a
chr19:54627979-54628968+	3	371/372/373	hetero	yes	chr19:53787677-53788770+	3	371a/372/373
chr3:162480895-162485756-	3	182/96/183	hetero	yes	chr7:129770405-129774988-	3	182/96/183
chr3:44449530-44450041-	3	25/93/106	hetero	yes	chr7:100093570-100094063-	3	25/93/106b
chr8:146049004-146055940-	3	939/1234/ 6849	hetero	yes	chr8:144394160-144400340-	3	939/1234/6849
chrx:149364083-149366356-	3	105-1/767/ 105-2	hetero	no	chrx:152392227-152394480-	3	105−1/105−2/767
chr14:9660229-9660537+	4	**6750**/**6749**/ 194-2/192	hetero	yes	chr11:64891158-64902450-	4	192/194−2/6750/ 6749
chr5:111988697-111989381-	5	367/302d/ 302a/302c/ 302b	hetero	no	chr4:112647876-112648547-	5	367/302d/ 302a/302c/ 302b
chr17:74024859-74025648+	6	17/18a/19a/ 20a/19b-1/92a-1	hetero	yes	chr13:91350617-91351382+	6	17/18a/19a/ 20a/19b-1/92a-1
chrx:131080800-131081707-	6	363-2/92a-1/ 19b-2/20b/ 18b/106a	hetero	yes	chrx:134169381-134170266-	6	363/92a-2/ 19b-2/20b/ 18b/106a
chrx:131455304-131461841-	6	450b/450a-1/ 450a-2/542/ 503/424	hetero	yes	chrx:134540193-134546701-	6	450b/450a-1/ 450a-2/542/ 503/424
chrx:142887921-142895601-	6	892c/890/ 888/ 892a/ 892b/891b	hetero	no	chrx:145992759-146001121-	6	892c/890/ 888/892a/ 892b/891b
chrx:48324406-48334989+	7	532/188/ 500a/362/ 501/660/502	hetero	yes	chrx:50003166-50014670+	8	532/188/ 500a/362/ 501/500b/ 660/502
chr7:166087997-166103699+	8	493/337/665/ 431/433/127/ 432/136	hetero	yes	chr14:100869074-100884771+	8	493/337/665/431/ 433/127/432/136
chr7:166241788-166285152+	42	379/411/299/ 380/1197/ 323a/758/ 329-1/329-2/ 494/1193/543 /495/ 376a-3/ 376c/376a-2/ 654/376b /376a/1185-2/ 1185-1/381/487b/ 539/889/544a/ 655/487a/382/ 134/668/485 /323b/154/ 496/377/541/ 409/412/ 369/410/656^a^	hetero	yes	chr14:101022070-101066786+	42	379/411/ 299/380/ 1197/323a/ 758/329-1/ 329-2/494/ 1193/543/495/ 376c/376a-2/ 654/376b/ 376a-1/ 300/ 1185-1/1185-2/ 381/487b/539/889/ 544a/655/487a/ 382/134/668/485/323b/ 154/496/377/541/409/ 412/369/410/656
chrx:144120330-144177313-	11	513a-1/513a-2/ 513a-3/506/ 507/508/ 514b/509-1/ 509-2/510/514a^a^	hetero	no	chrX:147225859-147284712-	11	513a-2/506/507/508/514b/ 509-2/509-3/509-1/510/514a-1/ 514a-2
chr19:54493931-54598758+	40	512/512-2 /1323/498 /515-2/519e/ 519e/519e /519c/1283/ 520ak/526b/519a-2 /525/523a/518f/ 519a/518b /526a-1/518c/524/517a/ 519d/518a-1/520g/ 518d-1/ 518g /518d-2/ 523b /516b-1/518a-3/ 517c/520g/519b/ 521/518e/ 518a-4/516a-2/ 516a-1/517b^a^	hetero	no	chr19:53666691-53762418+	46	512-1/512-2/1323/498/ 520e /515-1/519e/ 520f/515-2/519c/1283-1/520a/ 526b/519b/525/ 523/518f/ 520b /518b/526a-1/ 520c/518c/524/517a/519d/521-2/ 520d/517b/520g/516b-2/ 526a-2/518e/518a-1/518d/ 516b-1/518a-2/517c/ 520h /521-1/ 522/519a-1/ 527/516a-1/1283-2/ 516a-2/519a-2

**Notes.**

a MiRNA gene clusters with minor changes in cynomolgus macaques compared to their counterparts in humans.

b MiRNA gene clusters that newly identified in cynomolgus macaques. MiRNAs that have homologous sequences in macaque genome are marked by bold. Comparing the compositions of clusters in cynomolgus macaque to their orthologs in humans, differences in monkey and human are marked by underscore and double underscore, respectively.

hetero hetero-seed, miRNAs with distinct sequences in seed region homo homo-seed, miRNAs with identical sequences in seed region

**Figure 2:**
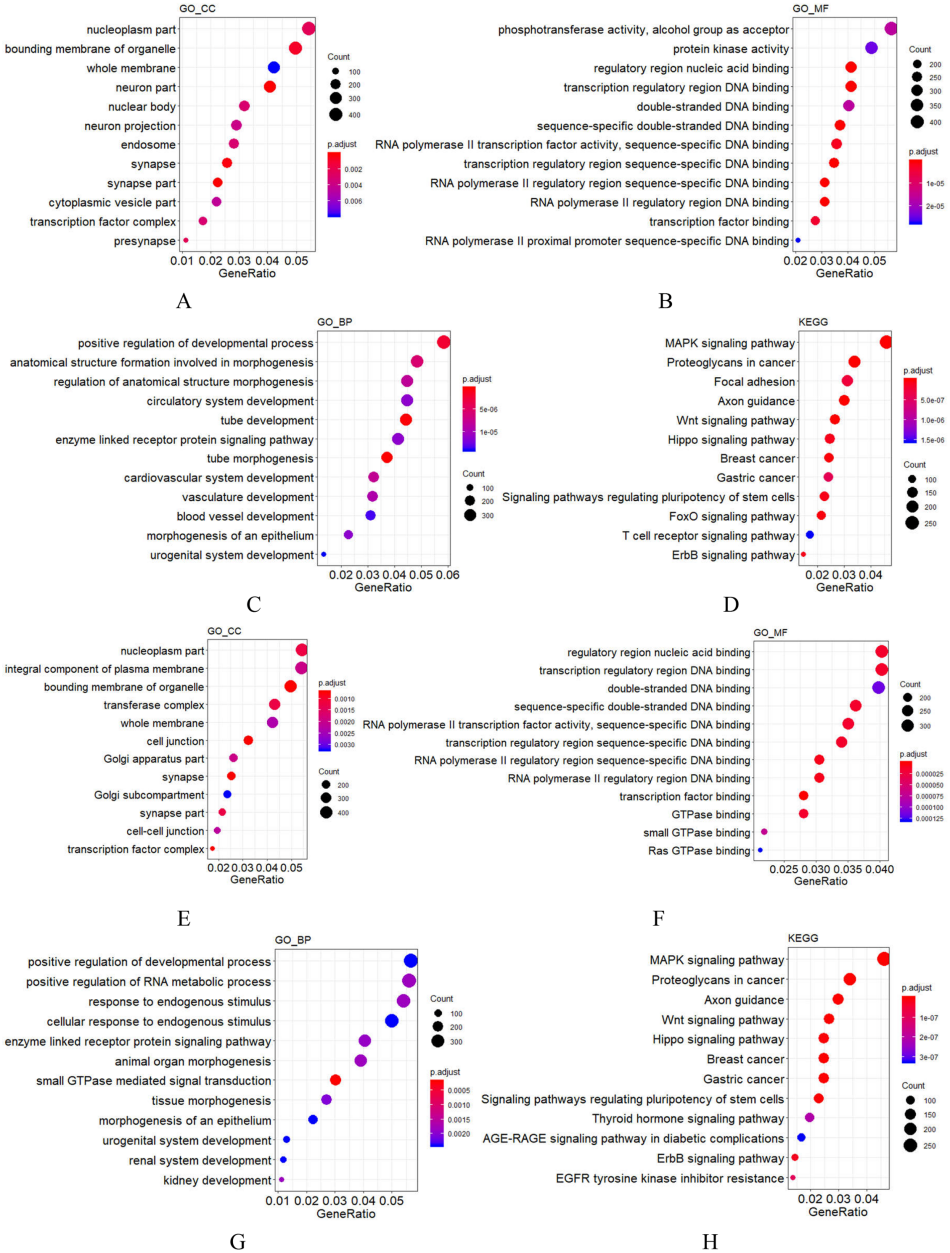
GO and KEGG analysis of mir-379/mir-656 (A–D) and mir-512/mir-517 (E–H) gene clusters. Vertical axis: GO terms and KEGG pathway names. CC, MF and BP represent cellular component, molecular function and biological process, respectively. The larger the point, the higher the degree of enrichment. The greater the number of candidate target genes in this GO term and KEGG pathway, the color of the points corresponds to different *p*-adjust ranges.

For most miRNA clusters revealed in this study, the number of pre-miRNAs ranged from two to three. Nonetheless, 10 large clusters with no less than five miRNA precursors were also observed (Table 3), which were composed of members from different miRNA families but with the same expression state. Among the 10 large clusters, most miRNAs in the six of them were detected by RNA-seq in this study, but none was detected by RNA-seq in the rest of the four clusters (Table 3). These results indicate that each of the 10 clusters was transcribed as a polycistronic element (Ryazansky, Gvozdev & Berezikov, 2011; Saini, Griffiths-Jones & Enright, 2007). Comparing the compositions of the 10 miRNA-clusters in cynomolgus macaques to their orthologs in humans, eight showed identical composition, whereas the mir-379/mir-656 and mir-512/mir-517 clusters showed minor changes. The mir-379/mir-656 cluster in macaques was positioned on chromosome 7 and contained 42 miRNA genes. Compared to humans, 40 out of the 42 miRNA genes were the same as humans. Simultaneously, a mir-376a copy was added and a mir-300 ortholog was lost. In the macaques, the mir-512/mir-517 cluster was located on chromosome 19 and contained 40 miRNA genes. Most shared common seed regions of the miR-515 family and likely originated from a common ancestor (Rodriguez et al., 2004; Zhang, Wang & Su, 2008). Comparing the miRNA composition of the mir-512/mir-517 cluster to the C19MC in humans, 38 of the 40 miRNA genes had orthologs in humans, two (mir-518g and mir-523b) were unique to macaques, and eight, including mir-522, mir-527, and six mir-520 variants, were not found in the macaque genome. These data show that although most C19MC members are conserved throughout the primate kingdom, frequent miRNA gains and losses, such as the gain of mir-518 and mir-523 copies and loss of mir-520 copies in macaques, have occurred (Berezikov et al., 2006; Iwama et al., 2013; Awan et al., 2017). These findings are consistent with previous results suggesting that the C19MC region is under rapid evolution (Iwama et al., 2013; Bentwich et al., 2005). Specifically, this region is enriched by dispersed Alu elements, which are believed to have facilitated the expansion of C19MC (Zhang, Wang & Su, 2008; Lehnert et al., 2009). To understand the functions of miRNA clusters, the potential targets of the two very large miRNA clusters were predicted using TargetScan, followed by GO functional annotation. Among the target genes of the mir-379/mir-656 cluster, those relevant to cellular components (CC) were enriched in synapse, membrane, and cell projection; those relevant to molecular function (MF) were mainly clustered in binding and catalytic activity; those relevant to biological process (BP) were mainly enriched in cellular process, biological regulation, metabolic process, development process, and response to stimulus (Fig. 2). For the mir-512/mir-517 cluster, the functional annotations of their target genes were similar to those of the mir-379/mir-656 cluster. KEGG analysis of the two very large miRNA clusters suggested that both regulate normal growth, development, and pathological processes through many pathways, such as MAPK signaling and Axon guidance (Fig. 2), consistent with previous studies on humans (Haller et al., 2010; Lavon et al., 2010; Cortez & Calin, 2009; Grün et al., 2005; Donker et al., 2012; Noguer-Dance et al., 2010; Tsai et al., 2009; Li et al., 2009a; Kleinman et al., 2014; Ward et al., 2014). The other eight large miRNA clusters in macaques are with conserved compositions to that in humans, indicating that their functions are also evolutionarily conserved.

## Discussion

The majority of reported cynomolgus macaque miRNAs are predicted based on homology to the rhesus genome (Veeranagouda et al., 2015). In this study, we firstly identified conserved miRNAs in cynomolgus macaques based on homology to all mammalian pre-miRNA sequences deposited in miRBase. To prevent mis-annotation of homologous sequences as miRNAs, we proposed a more stringent analysis process than previously reported (Veeranagouda et al., 2015). First, it requires that the full-length sequence similarity of homologous sequences exceed 93%. Secondly, it requires the homologous sequences to form a stable hairpin-like secondary structure with the mature sequences present in the stem. Next it also requires another supporting information: experimental supports for homolog in other species, or annotated as miRNA in other species, or reported in macaques previously. Using these criteria, 1,455 of the 1,697 homologous sequences were considered pre-miRNA gene loci of high confidence, more than half of which were firstly annotated in cynomolgus macaque genome (Yang et al., 2014; Veeranagouda et al., 2015). The data indicated that homology searching is a useful tool for high-throughput identification of conserved miRNAs (McCreight et al., 2017). Using these criteria, the false-positive annotations were reduced, but also some real pre-miRNA sequences were missed, such as poorly conserved miRNAs, or miRNAs that were not expressed at sufficient levels in the sequenced samples, or miRNAs that did not form acceptable hairpins. Thus, we just predicted a subset of conserved miRNAs in cynomolgus macaque. Using small RNA sequencing, 408 of the 1,455 genes were detected in peripheral blood leucocytes, which aided the confirmation of -5 p and -3 p mature miRNA sequences. The remaining 1,047 not supported with reads may be expressed in other tissues or under other circumstances, because large number of miRNAs exhibit noticeable spatiotemporal and tissue-specific expression. In addition, environmental stress or physiological disorders will significantly alter miRNA expression profiles (Pinhal et al., 2018). Therefore, we just experimentally verified a subset of miRNAs and further experimental confirmations of many miRNAs are needed in cynomolgus macaques.

When contrasting expression levels of miRNAs with their age, our results were in good agreement with previous reports that a positive correlation was found between sequence conservation and expression levels during miRNA evolution (Dannemann et al., 2012b). Newly emerged miRNAs are often expressed at lower levels compared with broadly conserved miRNAs (Wang et al., 2016), which suggest that miRNAs with low levels of conservation and expression may still be under evolutionary selection. Furthermore, the number and expression of miRNAs increased simultaneously during evolution, consistent with that observed in previous studies (Meunier et al., 2013; Berezikov et al., 2011; Lee, Risom & Strauss, 2007). The miRNA expansion may correlate with organismal complexity and body-plan innovation (Meunier et al., 2013; Christodoulou et al., 2010; Mohammed et al., 2018; Grimson et al., 2008).

We also systematically investigated the miRNA gene locations in the cynomolgus macaque genome. Unlike protein-coding genes, the locations of miRNA genes were highly polytropic. Most miRNAs resided in intergenic spaces or within introns of the hosts. Very few of miRNAs were found embedded in exons, overlapping-splice-sites, or antisense regions. Such unusual genomic arrangements of the miRNAs intensively raise our attention to explore their possible regulatory functions. For example, the *MIR1306* gene overlaps the DGCR8 coding region and the *MIR675* gene is embedded in the first exon of H19 lncRNA, both of which are conserved in primates (Morin et al., 2008; Friedländer et al., 2008; Keniry et al., 2012; Wang et al., 2018). It has been reported that the mir-1306 hairpin is cleaved by the Drosha-DGCR8 complex, which plays an important negative-feedback role in controlling DGCR8 expression (Morin et al., 2008; Friedländer et al., 2008; Keniry et al., 2012; Wang et al., 2018). *H19* is an important imprinted gene involved in placental development prior to birth, which limits the growth of the placenta by regulating miR-675 processes (Keniry et al., 2012; Wang et al., 2018; Han et al., 2009). Thus, it can be inferred that there is an expressive and functional interrelationship between miRNAs and host genes. For example, host genes can act as miRNA reservoirs and miRNA can negatively regulate its host. Among the exon-resident miRNAs identified in this study, it would be interesting to investigate whether such exonic miRNA hairpins serve dual roles as miRNA precursors as well as RNA instability elements as miRNA hairpin processing could destabilize host transcripts, as mentioned above. We also found some pre-miRNAs overlapping intron-exon junctions. These SO-miRNAs may be involved in regulating gene expression in cynomolgus macaques, whereby microprocessor complex-dependent cleavage of SO-miRNA exons could result in premature transcriptional termination of coding genes, as described in humans (Pianigiani et al., 2018). Two pairs of distinct antisense miRNAs were found in this study. The sequences and locations of miR-3120/miR-214 pair were conserved in mammals. Using miRNA target prediction software, we found that miR-3120-3p was predicted to interact with the 3′  UTR of heat shock protein family A member 5 (HSPA5) and miR-214-3p was predicted to target the 3′  UTR of phosphatase and tensin homolog (PTEN), consistent with previous research in humans (Scott et al., 2012). HSPA5 is a chaperone for protein folding in the endoplasmic reticulum and PTEN interacts with the mir-3120 host gene dynamin-3 to regulate synaptic proteins involved in receptor cycling and synaptic plasticity (Yang et al., 2008). The data suggest that the miR-3120/miR-214 pair represent a genetic unit in cynomolgus macaques that evolved to regulate the complex neuronal pathways associated with synaptic vesicle function and neuronal plasticity (Scott et al., 2012). For the miR-24-1/miR-3074 antisense miRNAs, although they have different seed sequences, KEGG analysis indicated that the targets of miR-24-3p and miR-3074-5p contribute to similar biological pathways, including Axon guidance and EGFR tyrosine kinase inhibitor resistance (Table S4). The miR-24-1/miR-3074 antisense miRNAs may regulate essential aspects of cellular function in coordination (Cordero et al., 2015). These overlapping regulatory functions indicate that direct or indirect coordinated regulation exist between these antisense miRNA pairs. Future research may identify other miRNAs resident in unusual genomic regions, such as exon-resident miRNAs, SO-miRNAs, and antisense miRNAs, which work in coordination with their host genes to regulate diverse and complex cellular functions.

Another characterization of miRNA genomic locations is that many are clustered together. In total, 78 miRNA clusters were found in this study, 68 of which were conserved in the human genome, including 10 large clusters with no less than five miRNA precursors. It was reported that these 10 large miRNA clusters have significant functions in humans. For example, miRNAs in the mir-17/92 cluster are potential human oncogenes (He et al., 2005) and critical regulators of normal development and disease, such as body height and cardiomyocyte proliferation (Chen et al., 2013; Bai et al., 2019). The X-linked mir-506/514 cluster has regulatory roles in testis development and spermatogenesis (Zhang et al., 2007). Furthermore, the very large mir-379/mir-656 and mir-512/mir-517 clusters both participate in development. The former, known as the chromosome 14 miRNA cluster (C14MC), is comprised of 42 miRNA genes in humans and is located at the DLK-DIO3 imprinted domain (Seitz et al., 2004; Morales-Prieto et al., 2013; Schmidt, Block & Golos, 2018; Sadovsky et al., 2015; Cavaillé et al., 2002; Glazov et al., 2008). The latter, known as the chromosome 19 miRNA cluster (C19MC), is comprised of 46 miRNA genes in humans and is imprinted in the placenta (Noguer-Dance et al., 2010). C14MC is reported to not only play important roles in the control of neurogenesis, embryonic development, and pregnancy, but is also associated with progression of hematopoietic and solid tumors (Haller et al., 2010; Lavon et al., 2010; Cortez & Calin, 2009; Grün et al., 2005; Donker et al., 2012; Morales-Prieto et al., 2013). In addition, variations in the expression of miRNAs in C19MC are associated with embryogenesis and tumorigenesis, suggesting a critical role of these miRNAs in development and cancer (Noguer-Dance et al., 2010; Tsai et al., 2009; Li et al., 2009a; Kleinman et al., 2014; Ward et al., 2014). Eight of the 10 miRNA-clusters in macaques are with conserved compositions to that in humans, indicating that their functions are evolutionarily conserved. The other two miRNA-clusters, the mir-379/mir-656 and mir-512/mir-517, showed minor changes to that in humans and then were used for functional prediction. The results indicated that both of the large miRNA-clusters regulate normal growth, development, and pathological processes through many pathways, consistent with previous studies on humans. Therefore, the miRNA clusters are important as they are involved in vital biological functions, including physiological and pathological processes, by targeting overlapping sets of genes cooperatively (Marco et al., 2013; Wang et al., 2016; Kim & Nam, 2006).

## Conclusions

In summary, 1,455 high-confidence miRNA gene loci were identified in the genome of cynomolgus macaques, 408 of which were also confirmed by RNA-seq. Due to the stringent annotation criteria and limited sequenced samples, we just obtained a subset of miRNAs in macaque. The miRNA genes in cynomolgus macaque were distributed in diverse locations. Very few of miRNAs, such as exon-resident miRNAs, SO-miRNAs, and antisense miRNAs, were predicated to regulate diverse functions in coordination with their host genes. Simultaneously, miRNAs processed from the large clusters were predicted to have essential cellular and developmental functions. This study not only expands the number of identified miRNAs in cynomolgus macaques but also provides clues for future research to improve our understanding of the evolution and function of miRNAs in primate. In the future, a great number of macaque miRNAs need to be verified by experiments. Further investigations on the functional role of primate-specific miRNAs are also needed because evolutionarily young miRNAs may correlate with organismal complexity and body-plan innovation and have the potential for development as novel biomarkers or therapeutic targets in complex diseases, especially in neuropsychiatric disorders.

##  Supplemental Information

10.7717/peerj.9347/supp-1 Supplemental Information 1 M. fascicularis pre-miRNA gene loci identified by homology searching °: underlined names indicate antisense miRNAs; MFE: minimal folding free energy; Category of age: The ages of the miRNAs were assessed based on the set of homologous sequences identified in other vertebrate species in miRBase. Vertebrates, Mammals, Primates and Cercopithecidae mean the miRNA homologous sequences were shared among Vertebrates, Mammals, Primates and Cercopithecidae, respectively. † : a: reported in cynomolgus macaques previously; b: annotated as miRNAs in other species; c: miRNA homologies in other species having experimental support.

10.7717/peerj.9347/supp-2 Supplemental Information 2 136 potentially novel mature miRNAs Average expression levels for each mature miRNA were normalized by transcripts per million; MFE: minimal folding free energy.

10.7717/peerj.9347/supp-3 Supplemental Information 3 383 M. fascicularis pre-miRNAs identified by small RNA sequencing °: single underlines means that the sRNA read perfectly matched the mature miRNA sequence deposied in miRBase, while the relavent pre-miRNA sequence did not found in cynomolgus macaque genome; Average expression levels for each mature miRNA were normalized by transcripts per million.

10.7717/peerj.9347/supp-4 Supplemental Information 4 KEGG analysis of mirror miRNA pair, miR-24-1/miR-3074 in cynomolgus macaque °: The same pathways that targets of miR-24-1 and miR-3074 contributed to are marked by underscore.

10.7717/peerj.9347/supp-5 Supplemental Information 5 Submission information for 50 mature miRNAs from 41 new pre-miRNA loci

10.7717/peerj.9347/supp-6 Supplemental Information 6 miRNA information for the four cynomolgus macaques

## Additional Information and Declarations

### DNA Deposition

The following information was supplied regarding the deposition of DNA sequences: The 73 new miRNA gene loci in cynomolgus macaques detected in this study were processed into 88 mature miRNAs. Mature miRNAs are available at the European Nucleotide Archive: LR594712-LR594765, LR745789-LR745838. This data is also available as a Supplemental File.

### Data Availability

The following information was supplied regarding data availability: The raw data are available as a Supplemental File.
